# Retrieval of negative autobiographical memories is associated with hostile attributions in ambiguous situations amongst people with schizophrenia

**DOI:** 10.1038/s41598-019-49058-4

**Published:** 2019-08-29

**Authors:** Tom J. Barry, José V. Hernández-Viadel, Dolores Fernández, Laura Ros, Jorge J. Ricarte, Fabrice Berna

**Affiliations:** 1 Department of Psychology, The University of Hong Kong, Hong Kong, Hong Kong; 2 Department of Psychology, The Institute of Psychiatry, Psychology & Neuroscience, King’s College London, London, United Kingdom; 3 Mental Health Service of Castilla-La Mancha, Cuenca, Spain; 4 Department of Psychology, Faculty of Medicine, University of Castilla-La Mancha, Albacete, Spain; 5 Hopitaux Universitaires de Strasbourg, 1 Place de l’Hopital, Strasbourg, Cedex France; 6 INSERM U-1114, 1 Place de l’Hopital, Clinique Psychiatrique, Strasbourg, Cedex France; 7 Université de Strasbourg, Faculté de Médecine, 4 rue Kirchleger, Strasbourg, France; 8 FMTS, Fédération de Médecine Translationnelle de Strasbourg, Strasbourg, France

**Keywords:** Human behaviour, Schizophrenia

## Abstract

Schizophrenia is characterised by difficulty understanding the thoughts and intentions of other people. Misunderstandings could lead people to attribute hostility to others’ actions. Theories suggest that we use our autobiographical memories to inform our understanding of other people but no study has examined the relation between memory and hostile attributions in schizophrenia. People with (*n* = 42) and without (*n* = 34) schizophrenia diagnoses completed The Ambiguous Intentions and Hostility Questionnaire (AIHQ) to assess their tendency to attribute hostility to other people’s actions and the Autobiographical Memory Test (AMT) to assess their ability to recall specific positive and negative autobiographical memories. In linear regressions the interaction between diagnostic group and the proportion of specific negative memories participants retrieved explained significant variance in each AIHQ index. Follow-up correlation analyses showed that participants with schizophrenia who retrieved more negative memories also attributed greater hostility to other people’s actions (*r* = 0.47) and reported that they would respond with greater aggression (*r* = 0.59). These correlations were in the opposite direction for controls. People with schizophrenia may use their memories for negative past events to understand the actions and intentions of other people, leading to attributions of hostility for otherwise benign actions.

## Introduction

Schizophrenia is characterised by problems with social cognition and, in particular, difficulty identifying and understanding the emotions, mental states and intentions of other people^1–5^. As current medications only have modest effects on social cognition and functioning, there is a need for investigations into the factors which contribute towards social cognitive difficulties in schizophrenia so that new targets for psychosocial interventions can be identified^6,7^. One such factor involves the ability to retrieve autobiographical memories, as both theories^8^ and research^9,10^ suggest that our understanding of other people is informed by our memories of past autobiographical events. However, there are few studies examining the nature of the association between autobiographical memory and aspects of social cognition within schizophrenia. In one daily diary study, Berna *et al*.^11^ found that, amongst people with schizophrenia, memories of social situations involving hostility were recalled frequently and with enhanced emotionality compared to non-hostile situations. If people with schizophrenia use these memories to inform their understanding of the actions of other people then this may explain why they exhibit a hostile attribution style^12^. However, no study has examined the relation between autobiographical memory and the tendency to attribute hostility to the actions of others amongst people with schizophrenia.

In order to understand the emotions and thoughts of others, people self-project into other people’s perspectives, simulating what they are feeling and thinking and imagining possible past events that might have caused them^9^. These simulations enable us to anticipate their future behaviour and plan our own behaviour accordingly. These inferences about other people, and the simulations that underlie them, are thought to be constructed from information stored in autobiographical memory^8,13^. When we are confronted with situations in which we must infer the mental states and intentions of other people we can draw from experiences from our past where we might have felt or behaved similarly and project these onto the person in the present^9^. This is particularly pertinent in schizophrenia where paranoia is common and where one might assume that other people are intentionally hostile towards us^3,12^. In such instances, when a person is confronted with a situation in which they feel they may have been wronged, such as when someone ignores them, they might retrieve memories of similar events where hostility was present, rather than retrieve other more benign or positive autobiographical memories. Moreover, those memories of hostility are more prone to be schematic, overgeneral, related in meaning to their common topic – hostility – and to lack the specific details making each stored experience unique^14^.

Indeed, there is considerable evidence that people with schizophrenia experience impaired autobiographical memory retrieval^15–17^ and that these impairments are correlated with their ability to generate and imagine possible future scenarios^18–23^. As such, when people with schizophrenia retrieve events from memory or imagine them, they are often experienced in a less vivid and personal manner compared to people without schizophrenia^24–27^. As autobiographical memory, future thinking and perspective taking are all thought to involve similar self-projection processes, and so also involve similar neural structures^8^, one might assume that people with schizophrenia would also differ from people who do not have schizophrenia in the way that they use their memories to simulate other people’s thoughts and behaviours. To our knowledge, only two studies have explored the association between autobiographical memory and the ability to understand the perspectives of other people^9,10^.

In these studies, across participants with and without schizophrenia, participants who were better able to recall autobiographical events were also better able to understand the mental states of other people^9,10^. In particular, Corcoran and Frith^9^ found that participants who retrieved more, and more detailed, autobiographical memories were better able to understand subtle hints made by other people and false beliefs and deception. Mehl *et al*.^10^ also found, using a similar index of autobiographical memory performance, that the ability to infer the intentions of another person was correlated positively with autobiographical memory abilities amongst people with schizophrenia but not control participants without schizophrenia. In addition, people with schizophrenia who were better able to recall autobiographical memories also performed better in a social interaction with a confederate (e.g., they maintained gaze, used proper modulation of speech tone etc.). Although these studies offer promising insights into the relations between autobiographical memory and social cognition, several questions remain.

First, it is of note that no study has yet examined the relation between autobiographical memory abilities and the tendency to attribute hostile intentions to other people, an important aspect of social cognition that is likely to contribute towards functional impairment^12^. Second, both Corcoran and Frith^9^ and Mehl *et al*.^10^ examined autobiographical memory performance in general. Existing work in this area suggests that it is retrieval of specific past events in particular – those lasting less than 24 hours – that informs the simulation of possible future events amongst people with schizophrenia^21^. However, no study has yet examined the association between recall of specific autobiographical events and social cognition. Finally, Corcoran and Frith^9^ scored the number of negative memories that participants retrieved, but they combined this with the number of memories that were odd/unrealistic. As such, no study has yet examined possible valence-specific effects in the relation between memory and social cognition. In the case of hostile attributions, it is reasonable to assume that people who are better able to retrieve memories for specific negative events may also be likely to make hostile attributions about other people, whereas the association between the ability to retrieve specific positive memories and hostile attributions may be less strong.

The present investigation therefore examined the associations between the ability to recall specific autobiographical events of positive and negative valence and the tendency to make hostile attributions. This tendency was measured using The Ambiguous Intentions and Hostility Questionnaire (AIHQ)^12^. In this task participants are presented with hypothetical scenarios in which another person appears to wrong them but where their intention is unclear (e.g., *someone cuts in front of you in the line at the grocery store*). Using this task, compared to diagnoses-free controls or people with low levels of paranoia, people with schizophrenia or high levels of paranoia typically attribute greater hostility to the actions of other people and blame them more, and based on these attributions, they report that they would respond in a more aggressive manner^3,12^. As such, the current investigation recruited participants with schizophrenia and compared them to a group of participants without schizophrenia. We hypothesised that participants who recalled more specific negative memories would show stronger attributions of hostility, especially given their problems recalling specific memories of positive or neutral valence^15,16^. However, for control participants, their ability to recall memories of any valence was expected to mean that their inferences would be weighted less heavily by the negative memories they retrieved. As such, we also hypothesised that the association between recall and hostile attributions would be strongest for participants with schizophrenia.

## Method

### Participants

Participants with neurological damage or disability, changes of medication within the past month or a history of epilepsy or alcohol or substance abuse were excluded from the study. People diagnosed by their current psychiatrist/psychologist as having schizophrenia (*n* = 42; Females = 26.2%) were recruited from the University Hospital Complex associated with the senior author. Upon recruitment into the study, participants diagnoses were confirmed by the senior author using the Mini-International Neuropsychiatric Interview (M.I.N.I.)^28^. Participants’ average age of disorder onset was 22.64 years (*SD* = 5.48) and the average disorder duration was 19.37 years (*SD* = 9.31). 97.6% (*n* = 41) of participants were taking antipsychotic medication at the time of the study, three of these were additionally taking anti-anxiety medication, 13 were additionally taking antidepressants and five were additionally taking both antidepressants and anti-anxiety medication. Control participants (*n* = 34; Females = 50.0%), who self-reported as never having been given a psychiatric diagnosis, were recruited from the local community near to the hospital complex.

### Measures

#### The ambiguous intention and hostility questionnaire (AIHQ)

The AIHQ^12^ presented participants with fifteen hypothetical negative social situations in which they are wronged by another person and they were instructed to imagine themselves in each situation. Participants were asked to report why they thought the event occurred. Their open-ended responses were later coded by the third author yielding scores from 1 (‘not at all hostile’) to 5 (‘very hostile’) for each situation. A total hostility index was then computed using the average score across the situations. Participants then answered three questions regarding the extent to which they thought the other person acted on purpose (from 1 ‘definitely no’ to 6 ‘definitely yes’), how angry it would make them feel (from 1 ‘not angry at all’ to 5 ‘very angry’) and how much they would blame the other person for these actions (from 1 ‘not at all’ to 5 ‘very much’). The scores for each of these questions were averaged for each situation and then a grand average across all situations was computed to yield a blame index. Finally, participants were asked how they would respond to the situation. Again, open-ended responses to this question were coded by the third author yielding scores from 1 (‘not at all aggressive’) to 5 (‘very aggressive’) which were averaged across the situations to compute an aggression index. To confirm the accuracy of the hostility and aggression codes, the fourth author coded a random selection of 30% of each of these responses and inter-rater reliability was strong (Hostility ICC = 0.96; Aggression ICC = 0.92).

#### The autobiographical memory test (AMT)

The AMT^29^ measured autobiographical memory specificity by presenting participants with five positive and five negative cue words in a fixed order (happy, sad, safe, evil, interested, awkward, successful, emotionally hurt, surprised and lonely). These words were selected from previous investigations of memory specificity amongst participants with diagnoses of schizophrenia^30–32^. For each word, participants were given 60 seconds to recall a specific memory. Verbal responses were transcribed, and memories were coded for whether they were specific (e.g., referring to a unique event lasting less than 24 hours) or non-specific (e.g., referring to an event that happened multiple times or over a prolonged period of time, or if they answered with a concept semantically related to the cue) or whether they neglected to respond at all (referred to as an *omission*). Each memory was additionally coded for whether they were positive in valence or negative or neutral^16,33–35^. In accordance with psychometric analyses of the AMT^36–38^, we quantified participants’ responses in terms of the proportion of specific responses that were given relative to the total number of cues minus the number of omissions each participant gave. A random selection of 40% of responses were second coded by the fourth author to confirm the accuracy of the original coding. There was a high level of agreement between coders (ICC = 0.96)

### Procedure

The study was approved by the Clinical Research Ethical Committee from the University Hospital Complex, CEIC12/2013, and was conducted in accordance with relevant guidelines and regulations. After receiving information about study aims, procedure, measures, voluntariness and confidentiality, participants and/or their legal guardians provided informed consent. The AIHQ was administered first, followed by the AMT.

### Data analysis strategy

Analyses were conducted using R version 3.5.2^39^. Following an analysis of between-group differences in participants’ characteristics, a 3 (Valence: Positive, Negative, Neutral) × 2 (Group: Schizophrenia; Controls) ANOVA tested for differences between groups in the proportion of specific memories recalled of each of positive, negative and neutral valence within the autobiographical memory test between participants with schizophrenia and controls. In this analysis a main effect of group was expected such that participants with schizophrenia had greater difficulty than controls retrieving specific memories irrespective of valence. Three multiple linear regressions were then performed, each predicting one of the AIHQ indices: hostility, blame and aggression. Each regression examined the unique associations between recall of specific memories of each valence and the dependent variable. As group differences in the relation between memory specificity and social cognition were expected, terms for the two-way interactions between group and mean-centred variables for the proportion of specific memories retrieved were also included in each regression. Where there was evidence of group differences in demographic characteristics (e.g., age) these variables were also included as predictors within the regression models. Within-group correlational analyses were used to follow-up any significant interaction effects within each regression.

## Results

### Between-group differences

There was a significant difference in the proportion of females between the group with participants with schizophrenia and the control group, *X*2(1) = 4.59, *p* < 0.032, *d* = 0.51, 95% CI[0.04, 0.97]. The groups did not differ significantly in their proportions of participants who completed university-level education (Schizophrenia = 11.9%; Controls = 14.7%), *X*2(1) = 0.92, *p* = 0.631, *d* = 0.22, 95% CI[−0.23, 0.67]. Participants with schizophrenia were significantly older (*M* = 42.02; *SD* 10.73) than controls (*M* = 32.79; *SD* 11.81), *t*(73) = 3.54, *p* < 0.001, 95% CI[4.04, 14.42], *d* = 0.82.

### Social cognition

Participants with schizophrenia reported significantly more anticipated hostility in the AIHQ (*M* = 1.91, *SD* = 0.71), compared to control participants (*M* = 1.36, *SD* = 0.36), *t*(74) = 4.07, *p* < 0.001, 95% CI[0.28, 0.81], *d* = 0.94, and reported that they would respond in a more aggressive manner (*M* = 1.59, SD = 0.45) than control participants (*M* = 1.36, SD = 0.34), *t*(74) = 2.35, *p* = 0.021, 95% CI[0.03, 0.41], *d* = 0.54. There was no significant difference between participants with schizophrenia (*M* = 2.83, SD = 0.66) and controls (*M* = 2.64, SD = 0.87) in their tendencies to attribute blame to the other person, *t*(74) = 1.05, *p* < 0.001, 95% CI[−0.17, 0.53], *d* = 0.24.

### Memory specificity

In the two-way ANOVA for group differences in the proportion of specific memories retrieved of each valence there were main effects of valence and group (see Table 1). The valence by group interaction was not significant. Pairwise comparisons of estimated marginal means showed that, across groups, compared to the proportion of neutral memories retrieved, participants recalled significantly more positive, *Mean Difference* = 0.14, *SE* = 0.02, *t*(222) = 6.09, *p* < 0.001, and negative memories, *Mean Difference* = 0.15, *SE* = 0.02, *t*(237) = 6.75, *p* < 0.001. However, there was no difference in the proportion of positive and negative specific memories that were retrieved, *Mean Difference* = −0.01, *SE* = 0.02, *t*(222) = −0.66, *p* = 0.788. Also, across valences, participants with schizophrenia retrieved significantly fewer specific memories compared to control participants, *Mean Difference* = −0.09, *SE* = 0.02, *t*(222) = −4.97, *p* < 0.001.

**Table 1:** Between-group differences.

	Means (SD)		ANOVA				
	Controls	Sz	Test	*df*	*F*	*p*	${{\eta }}_{{\rm{p}}}^{2}$
AMT							
Pos.	0.23 (0.15)	0.14 (0.15)	Valence	2, 237	26.43	<0.001	0.19
Neg.	0.27 (0.15)	0.13 (0.17)	Group	1, 237	24.72	<0.001	0.10
Neu.	0.07 (0.09)	0.03 (0.08)	Val. by Group	2, 237	2.39	0.094	0.02
Total	0.56 (0.28)	0.29 (0.30)					

Note. Means (and standard deviations) and mixed analyses of variance for group differences (Controls vs. Schizophrenia (Sz)) in the proportion of specific memories given in the autobiographical memory test (AMT) of positive, negative and neutral valences (val.).

### The relation between social cognition and memory specificity

Age and sex were included as predictors in the regression models predicting the AIHQ sub-scores given group differences in these variables. See Table 2 for regression findings.

**Table 2:** Linear regressions.

	Hostility				Blame				Aggression			
	b	SE	p	95% CI	b	SE	p	95% CI	b	SE	p	95% CI
Age	−0.018	0.005	**0.002**	−0.03, −0.01	−0.030	0.006	<**0.001**	−0.04, −0.02	−0.008	0.004	**0.039**	−0.02, −0.00
Sex	−0.240	0.124	0.057	−0.49, 0.01	−0.600	0.147	<**0.001**	−0.89, −0.31	−0.173	0.088	0.052	−0.35, 0.00
Group	0.863	0.137	<**0.001**	0.59, 1.14	0.504	0.162	**0.002**	0.18, 0.83	0.433	0.097	<**0.001**	0.24, 0.63
Prop. Pos.	−0.307	0.572	0.593	−1.45, 0.84	−0.581	0.673	0.391	−1.93, 0.76	0.161	0.403	0.691	−0.64, 0.97
by Group	−0.027	0.856	0.974	−1.74, 1.68	0.005	1.007	0.996	−2.01, 2.02	−0.586	0.602	0.334	−1.79, 0.62
Prop Neg.	1.873	0.515	<**0.001**	0.84, 2.90	1.095	0.606	0.076	−0.12, 2.31	1.465	0.363	<**0.001**	0.74, 2.19
by Group	−2.441	0.789	**0.003**	−4.02, −0.87	−2.781	0.928	**0.004**	−4.63, −0.93	−1.699	0.555	**0.003**	−2.81, −0.59
Prop Neu.	1.009	0.966	0.300	−0.92, 2.94	−0.436	1.137	0.703	−2.71, 1.84	0.888	0.680	0.196	−0.47, 2.25
by Group	−0.148	1.326	0.911	−2.80, 2.50	1.705	1.561	0.279	−1.41, 4.82	−0.041	0.934	0.965	−1.91, 1.82
	*R*^2^ = 0.51, *F*(9, 65) = 7.647, *p* < 0.001				*R*^2^ = 0.53, *F*(9, 65) = 7.999, *p* < 0.001				*R*^2^ = 0.45, *F*(9, 65) = 5.832, *p* < 0.001			

Note. Linear regression for the relations between the proportion of specific positive (Prop. Pos.), negative (Prop. Neg.) and neutral (Prop. Neu.) autobiographical memories retrieved and mean scores for the hostility, blame and aggression indices of social cognition. The main effect of diagnostic group (Controls vs. participants with schizophrenia) and interactions between specific memory retrieval and group are presented and age and sex are also entered as predictors in each regression. Significant (*p* < 0.05) findings are highlighted in bold.

Age explained significant amounts of variance in hostility, blame and aggression, and sex explained a significant amount of variance in blame, such that older age was associated with lower scores in each domain and being male was associated with lower blame scores in particular. Group explained a significant amount of variance in hostility and aggression and, unlike in the ANOVA, when other variables were accounted for group also explained a significant amount of variance in blame. The main effect for the proportion of negative specific memories retrieved explained a significant amount of variance in the hostility and aggression indices but not the blame index. However, further examination of the correlation coefficients across the whole sample showed that the relation between negative memory retrieval and the hostility (*r* = 0.04, *p* = 0.763) and aggression (*r* = 0.17, *p* = 0.134) scores yielded non-significant correlation coefficients. The proportions of positive and neutral specific memories retrieved and their interactions with group did not explain a significant amount of variance in any of the AIHQ sub-scores.

As hypothesised, the interaction between group and the proportion of negative specific memories retrieved, explained a significant amount of variance in each of the regressions. Also, each of the regression models explained a significant amount of variance in the respective dependent variables.

**Figure 1:**
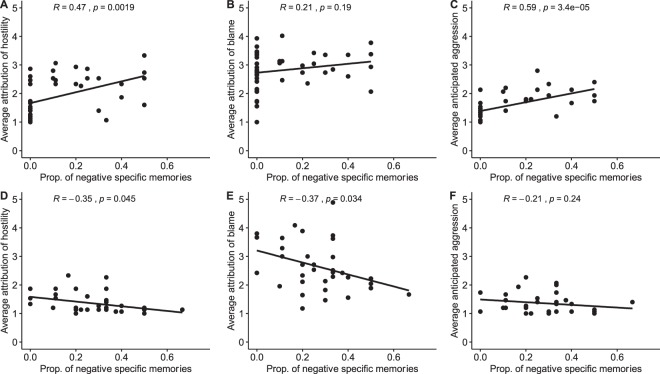
Scatter plots. Note. Scatter plots with lines of best fit and Pearson’s *r* coefficients for the relations between the proportion of negative specific memories retrieved and anticipated hostility, blame and aggression in social situations for participants with schizophrenia (**A**–**C**, respectively) and controls (**D**–**F**, respectively).

To explore the significant interactions in greater detail, correlational analyses examined the within-group associations between the proportion of negative specific memories retrieved and each of the AIHQ indices. For participants with schizophrenia, there was a significant positive correlation between the proportion of negative specific memories retrieved and perceived hostility (*r* = 0.47, *p* = 0.002) and anticipated aggression (*r* = 0.59, *p* < 0.001), but the correlation with blame was not significant (*r* = 0.21, *p* = 0.192). For control participants, there was a significant *negative* correlation between the proportion of negative specific memories retrieved and perceived hostility (*r* = −0.35, *p* = 0.045) and blame (*r* = −0.37, *p* = 0.034) but not aggression (*r* = −0.21, *p* = 0.240) (see Fig. 1).

## Discussion

Previous research suggests that social cognition is impaired amongst people with schizophrenia^2,3^ and that these impairments may be explained by problematic retrieval of autobiographical memories^9,10^. We provide the first investigation of the relation between autobiographical memory and the tendency to draw hostile attributions for the actions of other people.

In our regression analyses, as hypothesised, the interaction between the proportion of specific negative memories that participants could retrieve and whether or not they had schizophrenia explained a significant amount of variance in each of the AIHQ indices. Correlational analyses within each group suggested that participants with schizophrenia who retrieved more specific negative memories attributed greater hostility to the actions of the other person and also reported that they would respond with greater aggression. These correlations were also significant for control participants but were in the opposite direction. Control participants who retrieved more specific negative memories attributed less hostility to the actions of the other person and attributed less blame. These differential relations between memory and social cognition are similar to those found by Mehl *et al*.^10^ who found that the ability to infer the intentions of other people was correlated positively with autobiographical memory amongst people with schizophrenia but not controls.

For control participants, the scatter plots presented in Fig. 1 suggest that there may have been floor effects for their scores for the aggression index and as such there could have been insufficient variability between participants for any relation with autobiographical memory to emerge^9^. The negative correlation between negative specific memories and the blame and hostility indices may be because their ability to access both positive and negative specific memories to greater extent than their counterparts with schizophrenia may mean that they are able to use both of these memories to draw more benign or positive inferences. It may also be that the negative memories that participants with schizophrenia retrieved were also re-experienced with greater emotionality and intensity than control participants, as in previous investigations^14^, and so were likely to have a greater influence on their hostile attributions than was the case for control participants.

It is worth noting that the present investigation did not quantify to what extent participants responses in the AIHQ were based on their retrieval of memories of positive and negative events from their own past, or indeed to what extent the negative memories retrieved were associated with hostility or other negatively valenced content (e.g., memories of loss). Future investigations might additionally ask participants to retrieve autobiographical memories for each of the scenarios in the AIHQ (e.g.^40^) and examine the relation between the valence and specificity of these memories and their AIHQ responses.

Relatedly, our regression models only explained between 42–51% of the variance in each of the hostile attribution style indices suggesting that there may be other important variables that explain hostile attributions which were not included in the present analysis. Other aspects of social cognition, such as theory of mind, are likely to explain additional variance in participants’ hostile attributions^41^.

It is also worth noting that although theories suggest that autobiographical memories are used to simulate the thoughts and perspectives of other people, the correlational nature of our study precludes us from concluding that the retrieval of negative specific memories causes hostile attributions. Several other interpretations of our data are also possible and should be tested in future investigations. It could also be that participants who have experienced more hostile events in their life are more likely to retrieve memories for such events than participants who have experienced fewer such events. Indeed, it is possible that the experience of negative life events amongst participants with schizophrenia, and particularly those events in which one experiences hostility, may provide a better explanation for the group differences in hostility than negative memory specificity did. An analysis of preceding life stressors to subsequent AIHQ performance is therefore warranted. It is worth noting also that, where one has experienced significant hostility throughout one’s life it may benefit their survival if they tend to infer hostile intent in the actions of other people and so respond to such intentions accordingly. Nevertheless, it is probable that if such a bias perseveres even when hostility is no longer present in one’s life, then this may become maladaptive. Biased retrieval of negative specific memories may be one factor that leads to the perseverance of this hostile attribution style.

Also, given the social functioning impairment that accompanies social cognitive difficulties such as hostile attributions^1^ it may also be that the reason people with a hostile attribution style are better able to recall negative memories is because their hostile attribution style leads them to create hostile or negative situations in their lives and so there are many such situations that they can later retrieve when completing measures like the AMT. It could also be that due to other persecutory biases and anxiety, people with schizophrenia are particularly prone to falsely remembering events in which others acted in a hostile manner, and to do so in a highly schematic and general manner^14^. Again, future longitudinal analyses, should examine the causal order of negative life events, negative memory retrieval and hostile attributions. In particular, future studies could also use existing paradigms for encouraging the retrieval of specific memories^42,43^ but adapt them such that participants retrieve negative or hostility-related specific memories. The effects of this manipulation, and its possible interaction with schizophrenia symptoms, on hostile attributions could then be examined.

Nevertheless, the findings presented here extend those of two previous studies regarding the relation between autobiographical memory and social cognition in people with schizophrenia^9,10^. Our findings suggest that autobiographical memory is not only related to differences in theory of mind and understanding social hints and signals sent by other people^9,10^ but that it is also related to another important aspect of social cognition in schizophrenia, hostile attributions. Also, previous studies in this area have not explored valence-specific effects and have instead found that biases in autobiographical memory are associated with social cognition impairments. Our findings suggest that the ability to retrieve negative memories in particular is associated with one’s understanding of other people’s apparently hostile intentions. Finally, unlike previous investigations that quantified individual differences in autobiographical memory using broad indices of how many memories one could retrieve, specific or otherwise^9,10^, our findings suggest that variability in the ability to retrieve specific events from one’s past in particular, are related to social cognition. This finding aligns with a vast amount of other research suggesting that this ability is crucial for emotion regulation and planning and hoping for the future^44^ and research suggesting that such memories allow us to plan solutions for social problems that we encounter^45,46^. Future research should examine the relation between memory specificity and other aspects of social cognition.

Relatedly, our investigation replicated the findings of other research regarding the impairment in autobiographical memory specificity observed amongst people with schizophrenia^15–17^. Existing psychosocial interventions for social cognitive problems in schizophrenia do not yet include components for improving autobiographical memory^6,7^. Future studies could examine the augmentative effects of combining aspects of Memory Specificity Training (MeST)^47–50^, and particularly training people to retrieve specific positive memories, within existing social cognition interventions.

Our investigation also partially replicated the findings from the recent Social Cognition Psychometric Evaluation (SCOPE) study regarding hostile attribution styles amongst people with schizophrenia^3^. In this previous investigation and the investigation reported here, participants with schizophrenia attributed greater hostility to the other person’s actions than controls did. However, for the other AIHQ indices some mixed findings emerged. In the SCOPE investigation but *not* the present investigation, participants with schizophrenia also attributed greater blame to the other person than controls did. Conversely, in the present investigation but *not* the SCOPE investigation, participants with schizophrenia reported that they would respond in a more aggressive manner to the actions of the person in the scenarios, compared to controls. There are several possible reasons for these discrepancies.

It is worth noting that the SCOPE investigation found that the AIHQ task showed poor reliability. However, Pinkham *et al*.^3^ concluded that as the tendency to interpret and respond to other people as though they are intentionally hostile has important functional implications for people with schizophrenia and there are few, if any, validated alternatives to the AIHQ^51^, further research in this area is therefore needed. Since the SCOPE study was first designed newer alternatives have emerged, such as the judgement of intention task developed by Buck *et al*.^52^ or the interview-based assessment of hostile attributions from the Observable Social Cognition: A Rating Scale (OSCARS)^53^ that could prove useful in future replications of the effects found here.

The discrepancy between the findings presented here for group differences in AIHQ scores and other similar investigations such as the SCOPE study could also be due to participant characteristics. Participants in the schizophrenia groups in both investigations were otherwise similarly aged (approximately 40 years), had similar age of disorder onset (approximately 22 years), both included proportionally more males than females, and in both studies participants were mostly taking anti-psychotic medication. Previous research has shown that AIHQ performance differs as a function of individual differences in paranoia^12^ and so it is possible that the discrepancy between studies can be attributed to differences in participants’ paranoia. Future investigations could examine the relations between individual differences in paranoia and related aspects of psychosis – perhaps measured using scores from the Positive and Negative Syndrome Scale – and performance in the AIHQ and autobiographical memory specificity.

In conclusion, the present investigation further contributes to our understanding regarding the relations between autobiographical memory and social cognition in people with schizophrenia. In particular, participants with schizophrenia who recalled more specific negative memories also attributed greater hostility to the actions of another person and reported that they would respond to these actions with greater aggression. These findings suggest new targets for social cognitive impairments in schizophrenia in the form of memory therapeutics.

## Data Availability

The datasets analysed within the current study are available from the corresponding author on reasonable request.
